# Two-stage treatment for severe spinal kyphotic deformity secondary to tuberculosis: halo-pelvic traction followed by a posterior-only approach correction

**DOI:** 10.1186/s12891-022-05974-7

**Published:** 2022-11-18

**Authors:** Longtao Qi, Yao Zhao, Beiyu Xu, Chunde Li, Yu Wang

**Affiliations:** Department of Orthopaedics, Peking University First Hospital, Xicheng District, Beijing, 100034 China

**Keywords:** Spinal tuberculosis, Halo-pelvic traction, Severe spinal deformity

## Abstract

**Background and purpose:**

Several surgical procedures are used to treat tuberculous kyphosis. However, the treatment of extreme spinal kyphosis is challenging, and associated with various complications. Halo traction has been used as an adjunctive method in the treatment of severe spinal deformities. However, there are few reports about the effectiveness of halo-pelvic traction (HPT) for the treatment of extreme spinal kyphosis secondary to tuberculosis. This study evaluated the effectiveness of halo-pelvic traction followed by a posterior-only approach correction in the treatment of severe spinal kyphosis secondary to tuberculosis.

**Methods:**

The records of 19 patients with severe spinal kyphosis secondary to tuberculosis were retrospectively reviewed. All 19 patients were treated with a two-stage approach: HPT combined with posterior fusion surgery by a posterior-only approach. Radiographic parameters were measured and evaluated. America Spinal Injury Association grade (ASIA), Scoliosis Research Society outcome (SRS-22) score, and complications were also evaluated.

**Results:**

There were 9 males and 10 females, with an average age of 29.7 years at the time of surgery. The average HPT duration was 10.4 weeks. The mean kyphosis angle decreased from 131.40 ± 10.7° pre-traction to 77.1 ± 7.4° post-traction (*P* < 0.01). The traction correction rate was 41.3%. The mean postoperative kyphosis angle was 65.7 ± 8.5°, and the surgical correction rate was 8.7%. Of the total correction, 82.6% was the result of HPT. At a mean follow-up of 26.5 months, the average kyphosis correction loss was 2.9°. The mean sagittal balance was 11.1 ± 45.2 mm before traction, − 25.0 ± 37.4 mm after traction, 7.0 ± 13 mm after surgery, and 2.8 ± 9.6 mm at the final follow-up. The mean preoperative SRS-22 score was 3.0 and postoperative was 4.2 (*P* < 0.01). The neurological status of most patients was improved. The total complication rate was 15.7%, including 1 neurological and 2 non-neurological complications.

**Conclusions:**

HPT is effective in the management of severe spinal kyphotic deformity secondary to tuberculosis. Preoperative HPT can greatly reduce global kyphosis, and the need for corpectomy.

**Supplementary Information:**

The online version contains supplementary material available at 10.1186/s12891-022-05974-7.

## Introduction

Despite the success tuberculosis drug treatment, 3 to 5% patients with spinal tuberculosis develop a kyphosis of > 60° [1, 2]. This can cause the cosmetic, psychological, cardio-respiratory, and neurological problems, especially when the kyphosis is severe (> 90°) [2–4]. Patients with kyphosis > 60° always need surgery to relieve spinal cord compression and treat neurological complications, back pain and dysfunction, and poor sagittal balance [2, 5, 6]. Several different surgical procedures are used to treat the tuberculous spinal kyphosis. However, these procedures are challenging because a complex correction and osteotomy are always required, and there is a high rate of complications such as neurological injury and vascular injury [5, 7–13].

In the 1970s, O’Brien et al. introduced the use of halo-pelvic traction (HPT) for patients with spinal deformities, and reported impressive correction of the deformities [14]. In this study, we evaluated the safety and effectiveness of HPT combined with posterior fusion surgery by a posterior-only approach for the treatment of patients with severe spinal kyphosis secondary to tuberculosis.

## Methods

### Ethics statement

This study was approved by the Ethics Committee of our Hospital. All patients provided consent for all procedures performed.

### Patient characteristics

The records of 19 patients with severe spinal kyphosis secondary to tuberculosis who were treated with HPT combined with posterior fusion surgery by a posterior-only approach were retrospectively reviewed. All of the surgeries were performed by the senior author between 2017 and 2020. Clinical and radiographic data were collected and evaluated by an independent spine surgeon who was not involved in the any patient’s care. Patients who met the following criteria were included in the analysis. (1) Severe spinal kyphosis secondary to tuberculosis (kyphosis angle ≥100°). 2) The spinal tuberculosis was cured or silent. (3) Patient was treated with HPT combined with a posterior-only approach for surgical correction. Patients with kyphosis due to other reasons, such as congenital, idiopathic, or traumatic factor, or a history of spine surgery, or osteoporosis, or age > 45 years were excluded. Demographic data collected included age, sex, and duration of traction.

### HPT protocol

**Fig. 1:**
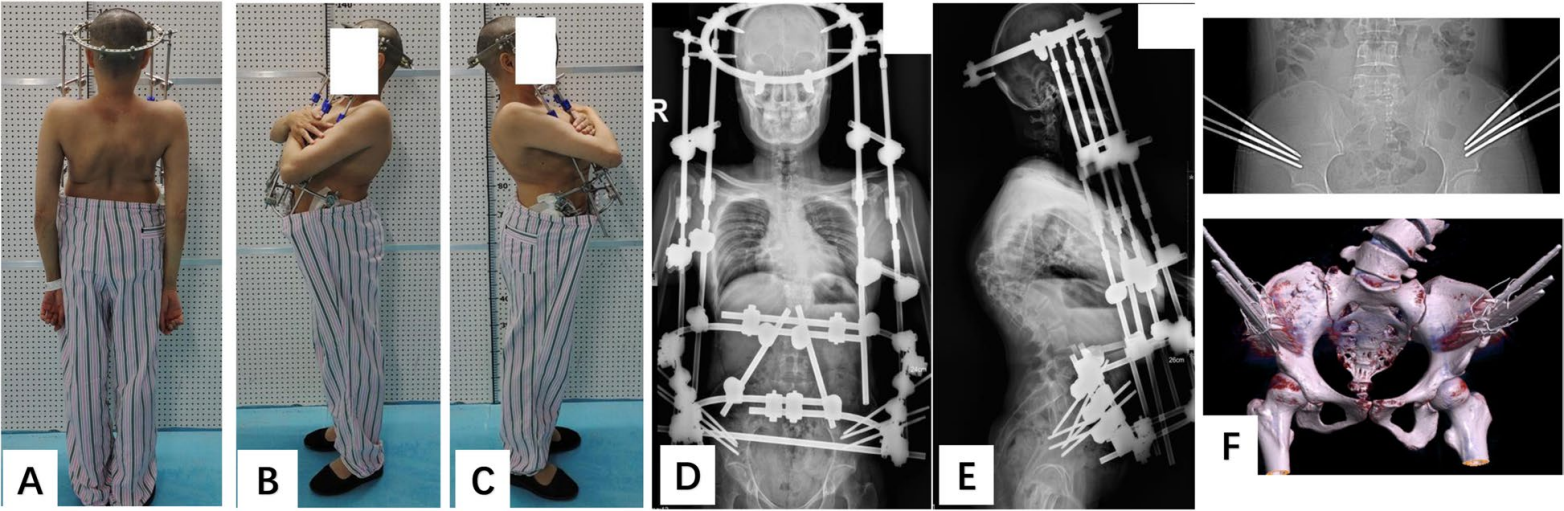
Modified halo-pelvic traction. Posterior (**A**) and lateral (**B**, **C**) view photographs. Standing anteroposterior (**D**) and lateral radiographs (**E**). Photographs of the bony pelvis with pelvic pins (**F**)

For the first stage, HPT was administered with a modified halo-pelvic apparatus as described in our previous report [15]. The HPT device consists of a head ring, a pelvic ring, and retractable connecting rods, and was applied under general anesthesia. Briefly, 3 pelvic pins are inserted into the area between the inner and outer table of the ilium on each side, and drilling through the iliac crest is not required (Fig. 1F). The number of pins is based on the patient’s bone mineral status and anatomical variation. The skull device is then placed, as previously reported [16]. Different from traditional HPT, the pelvic ring is a half-ring and all the rods are placed on the anterolateral side of the truck (Fig. 1). The frame is distracted at a rate of 0.5 cm per day for the first week, and then the distraction rate is decreased to 0.3–0.5 cm every 2–3 days. Patients received daily neurological examinations, and if any neurological complications occurred the traction length was reduced to the previous length.

### Surgical procedure

For the second stage, posterior spinal correction and fusion surgery was performed after 2–4 months after the beginning of traction. Pedicle screws were placed at the levels of fixation using an intraoperative navigation system. The choice of osteotomy depended on the magnitude and location of the deformity, neurological deficits, the desired correction, and the sagittal alignment. If necessary, two rods were placed on each side through the connector to stabilize the correction. Abundant bone graft was placed after posterior correction to promote fusion of the posterior column. Somatosensory evoked potentials (SEP) and motor evoked potentials (MEP) were used to evaluate spinal cord function during surgery. After surgical correction, the HPT device was removed. Patients were instructed to wear an orthosis for 3 months after surgery.

### Radiographic and clinical evaluation

Radiographic parameters examined included the major kyphotic curve angle and the sagittal vertical axis (SVA), and were measured from biplanar, full-standing, standard radiographs of the whole spine obtained pre-traction, post-traction, and post-operative. During traction, radiographs were taken every 2 weeks. All radiographic measurements were performed by two independent observers, and an average of 2 values was used for analysis.

Patient pain, neurological status, and daily functional activity were assessed using the Scoliosis Research Society-22 (SRS-22) instrument and the America Spinal Injury Association (ASIA) instrument preoperatively and at follow-up. Complications, including instrumentation failure, neurological deficits, and revision surgery were also examined.

### Statistical analysis

Data were presented as the mean ± standard deviation, and comparisons of radiographic measurements and clinical outcomes were performed using a parametric paired t-test. Values of *P* < 0.05 were considered to indicate a statistically significant difference. All analyses were conducted using SPSS version 20.0 software (SPSS Inc., Chicago, IL, USA).

## Results

Of the 19 patients, 10 were female and 9 were male and the mean patient age at the time of surgery was 29.7 years (range, 17–38 years). The mean traction duration was 10.4 weeks (range, 8–14 weeks). Patient demographic data are summarized in Table 1.

**Table 1:** Demographic characteristics and operative details

Parameter	Descriptive statistics
Gender	
M	9
F	10
Age (yr) ^a^	29.7 ± 6.0 (17–38)
Main symptom	
Persistent back pain	8
Neurological deficit	10
Respiratory dysfunction	7
Number of bodies involved^a^	4.6 ± 1.1(3–7)
Location of apex of the deformity	
Thoracic (T1–T10)	12
Thoracolumbar (T11–L2)	4
Lumbar (L3–L5)	3
Period of traction (weeks) ^a^	10.4 ± 1.9 (8–14)
The blood loss (ml) ^a, b^	558 ± 171 (300–850)
Operative time (min) ^a, b^	251 ± 52 (180–402)
Osteotomy^b^	
PSO	4
PVCR	0
Period of follow-up (months) ^a^	26.5 ± 7.0 (20–42)

^a^The values are represented as mean ± SD

^b^The details of the posterior spinal correction and fusion after HPT

*PSO* Pedicle subtraction osteotomy, *PVCR* Posterior vertebral column resection

There was a significant improvement of the major kyphosis angle after HPT (Table 2). The major kyphosis angle decreased from a mean pre-traction value of 131.40 ± 10.7° (range, 109°-148°) to a post-traction value of 77.1 ± 7.4° (range, 57°-87°) (*P* < 0.01). The traction correction rate was 41.3%. After the surgery, the mean kyphosis angle was 65.7 ± 8.5° (range, 45°-80°), and the surgical correction rate was 8.7%. Of the mean total correction, 82.6% of the correction was due to HTP traction. The mean follow-up duration was 26.5 months, and the mean kyphosis correction loss was 2.9° at the final follow-up. The pre-traction SVA was 11.1 ± 45.2 mm (range, − 98 mm – 80 mm), and the post-traction SVA was − 25.0 ± 37.4 mm (range, − 138 mm – 12 mm) (*P* < 0.001). The mean SVA after surgery was significantly different from that after HPT (*P* < 0.001), but similar to the SVA before HPT (*P* = 0.343).

**Table 2:** Radiographic parameters of the patients with severe kyphotic deformity secondary to spinal tuberculosis (n = 19)

Parameter	Pre-traction	Post-traction	Post-surgery	Follow-up
Kyphosis angle (°)	131.4 ± 10.7	77.1 ± 7.4^*^	65.7 ± 8.5^*#^	68.6 ± 9.4^*#^
SVA (mm)	11.1 ± 45.2	−25.0 ± 37.4^*^	7.0 ± 13^#^	2.8 ± 9.6^#^

Note: * Compared with pre-traction data, *p* < 0.05. # Compared with post-traction data, *p* < 0.05

The mean pre-traction and final follow-up SRS-22 scores are shown in Table 3. At the final follow-up, the total SRS-22 score and all individual domain scores were significantly improved from those pre-traction (P < 0.001). Patient neurological status as determined by ASIA scores are shown in Table 4. Of the patients with preoperative neurologic deficits, one recovered to ASIA D and the rest improved to ASIA E after surgery. No patients experienced neurologic deterioration in the immediate postoperative period.

**Table 3:** Comparison of SRS-22 results before HPT and at follow-up (*n* = 19)

SRS-22 domain	Pre-traction (Mean ± SD)	Follow-up (Mean ± SD)	*p* value
Function	3.0 ± 0.3	4.1 ± 0.3	< 0.001
Pain	3.1 ± 0.3	4.3 ± 0.2	< 0.001
Self-image	2.8 ± 0.3	4.1 ± 0.3	< 0.001
Mental health	3.0 ± 0.2	4.1 ± 0.2	< 0.001
Sub total	3.0 ± 0.2	4.2 ± 0.2	< 0.001
Post treatment satisfaction	NA	4.4 ± 0.4	NA

*NA* not applicable, *SRS-22* scoliosis research society outcomes questionnaire

**Table 4:** Neurological status of the patients (ASIA) (*n* = 19)

ASIA grading	Pre-traction	Post-traction	Post-surgery	Follow-up
A	0	0	0	0
B	0	0	0	0
C	3	2	2	0
D	7	3	2	1
E	9	14	15	18

Two patients had complications (one patient had 1 complication, and the other had two complications), and the overall complication rate was15.7% (3 complications in 19 patients). The muscle strength of the right leg of one patient decreased after 6 weeks of traction, and recovered after the traction length was adjusted to the previous length. In one patient a pelvic pin became loose after 10 weeks of traction, and corrective surgery was performed 2 days later. At 3-month follow-up, pull-out of a proximal screw was noted. Revision surgery was not required, and there was no progression of the screw pull-out during the following 2 years. No screw malposition occurred in any patient. Some patients had cervical discomfort during traction, but this symptom resolved after surgery. There were no infections, or other complications observed.

## Discussion

Spinal tuberculosis often involves and destroys multiple adjacent vertebral bodies resulting in progressive worsening of kyphosis. The resulting severe kyphosis can result in psychological, cardio-respiratory, and neurological problems, especially when the kyphotic deformity is > 90° [2–4]. Of the patients in this study, 53% had neurologic deficits, 37% respiratory insufficiency, and 42% back pain. For patients with a severe kyphotic deformity, surgery is always need to relieve spinal cord compression and to treat neurologic complications, back pain and dysfunction, and poor sagittal balance [2, 5, 6].

Surgical approaches for treating tuberculous kyphosis include anterior, anterior–posterior combined, and posterior-only approaches. Anterior-only procedures were the first used to treat tuberculous kyphosis [17, 18]. However, they resulted in minimal correction of kyphosis with a high rate of complications [18]. The anterior–posterior combined approach resulted in better correction, but 2 separate operations were required and there was a high complication rate, long operative duration, large blood loss, and high morbidity rate [5, 6, 19, 20]. A posterior approach allows spinal cord decompression and fusion in the same operation, and minimizes the risk of injury to vascular structures and viscera. With a posterior-only procedure, a posterior osteotomy such as the Smith-Petersen osteotomy (SPO), the pedicle subtraction osteotomy (PSO), and the posterior vertebral column resection (PVCR) is often used to address regional kyphosis. However, because typically multiple adjacent vertebral bodies have been destroyed and fused together, the SPO is not very suitable for rigid and severe tuberculous kyphosis. Because a PSO obtains 2.5° of correction per mm of posterior closure and thus excessive shortening of the spine cord may increase the risk of neurologic injury, it has been suggested that PSO should only be used to treat kyphosis < 40° [12, 13, 21, 22]. PVCR was descried by Suk et al. [11], and is reserved for more severe cases of kyphosis. PVCR can produce a correction up to 50% of sagittal plane in patients with severe tuberculous kyphosis [9, 10, 12, 13, 23, 24]. However, PVCR is a challenging procedure to perform and is associated with a high risk of neurologic and vascular injuries.

A summary of the major outcomes of surgery for tuberculous kyphosis via a posterior-only approach reported by different studies are shown Table 5. The major kyphotic angle of patients in our study was greater than that in reports by Rajasekaran et al. [9, 10, 12, 13, 23–25]. Additionally, the mean number of vertebral bodies involved in our study, as determined by computed tomography (CT) was 4.6. For such severe, rigid kyphotic deformities it is very difficult to access to the apex of the deformity through an anterior approach with complications, and the PSO and SPO cannot provide sufficient correction for severe kyphotic deformities. Furthermore, in patients with severe kyphosis the nerve roots of the major kyphosis area are squeezed into a very small region which makes performing a vertebral osteotomy and mesh insertion (VCR) very difficultly through a posterior approach.

**Table 5:** Comparisons to the studies through posterior-only approach for post-tubercular healed kyphotic spinal deformity^a^

Author and year	Number of patients	Age (years)	Follow-up (months)	Type of osteotomy	Kyphosis (°) (preoperative/final follow-up/correction loss)	Functional core (preoperative/postoperative)	Complications (%) overall/neurological
Liu et al. [9] 2016	28	20.9	96.9	PVCR	70.7 / 30.2 / 8.5	SRS-22: 2.61 / 3.68 ODI: 55.1 / 15.0	21.4 / 3.5
Rajasekaran et al. [10] 2010	17	18.3	43	CWO	69.2 / 32.4 / 5.4	VAS: 9.2 / 1.5 ODI: 56.4 / 10.6	29.4 / 5.88
Zhang et al. [24] 2013	15	35.8	36.1	PVCR	92.3 / 34.5 / 2.4	VAS: 8.7 / 2.2 ODI: 46.5 / 5.7	NA / 0
Garg et al. [13] 2021	47	16	43	PVCR	68.2 / 30.9 / 1.3	SRS-22: 2.7 / 4	29.7 / 8.5
Zeng et al. [12] 2012	36	34	31.3	PSO (7) / PVCR (29)	89.3 / 29.3 / NA	VAS: 2.0 / 0.7	16.6 / 8.3
Wang et al. [23] 2009	9	26.2	30.6	PVCR	97.2 / 17.2 / 1.6	NA	11.1 / 11.1
Muheremu et al. [25] 2017	40	33	range 2–19	HPT + PF	95.6 / 28.3 / NA	NA	NA / NA
Current study	19	29.7	26.5	HPT + PF	131.40 / 65.7 / 2.9	SRS-22: 3.0 / 4.2	15.7 / 5.3

*NA* not applicable, *SRS-22* scoliosis research society outcomes questionnaire, *ODI* Oswestry Disability Index, *VAS* visual analog scale, *PVCR* posterior vertebral column resection, *CWO* closing opening wedge osteotomy, *PSO* pedicle subtraction osteotomy, *HPT* halo-pelvic traction, *PF* posterior fusion

^a^Only studies with 5 or more cases have been considered

The 19 patients in this report all received HPT prior to correction via a posterior-only approach. There was a significant correction of the major kyphosis of 54.3° (41.3%) after HPT as compared to prior to HPT. Of the mean total correction of all patients (65.7°/50%), 82.6% was the result of HPT. It is much safer to perform surgery with a kyphosis of around 77° than when it is > 131°. The reduction of kyphosis angle prior to surgery is an advantage of HPT. The reduction of kyphosis prior to corrective surgery reduces the difficulty of the corrective surgery and alleviates the need for osteotomy. Of our 19 patients, 4 patients received a PSO and no patients received PVCR. The mean surgical blood loss in our 19 patients was 558 ± 171 ml (range, 300–850 ml); a value much lower than that reported by Wang et al. [23] (2933 ml) and Zhang et al. [24] (1653 ml), Rajasekaran et al. [10] (820 ml) and Karla et al. [22] (940 ml).

In our patients, the SVA was located further behind the sacrum after HPT; however, sagittal balance was restored after surgery. Thus, the use of HPT has no negative impact on sagittal balance. Of the patients with neurologic involvement preoperatively, one recovered to ASIA D and the rest all improved to ASIA E. Notably, the main improvement of neurologic involvement was due to HPT. This may be related to the spinal cord and its blood vessels slowly adjusting to the increased length of the spinal canal during the period of traction, and this may reduce the rate of, or avoid spinal cord injury. For patients who had pulmonary impairment preoperatively, breathing difficulty was completely relieved during follow-up. This confirms that HPT helps to improve pulmonary function.

**Fig. 2:**
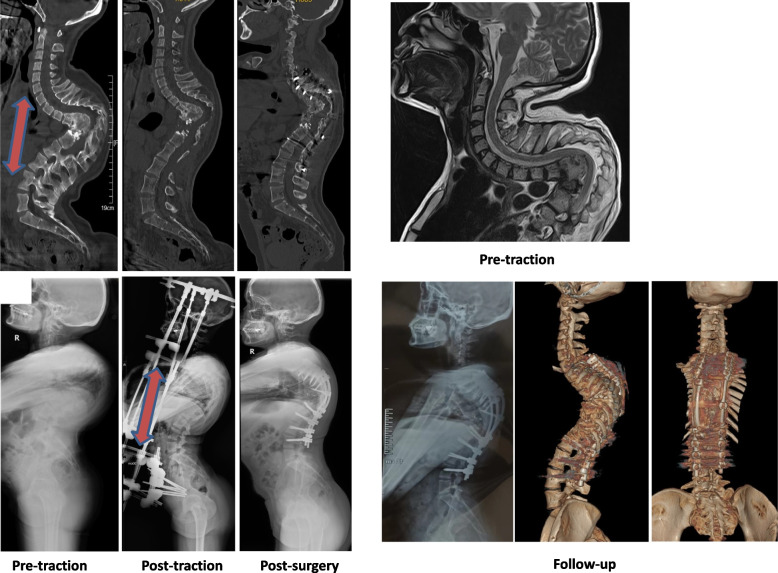
A 31-year-old male presented with a 17-year history of tuberculous angular kyphosis. The patient complained of back pain, difficulty breathing, and weakness of both lower extremities (ASIA D). Preoperative CT demonstrated extensive fusion of the vertebral bodies from T6-T12, and MRI showed the spinal cord had been drawn into the angular kyphosis region. After 12 weeks of HPT, the kyphosis angle decreased from a pre-traction value of 145° to a post-traction value of 87°. CT showed an abruption at the distal end of the kyphosis (between L1 and T12); however, neurological symptoms did not become worse. Posterior decompression, pedicle screw fixation (T1-L4), PSO, and autologous bone grafting were performed via a posterior approach to achieve final correction. Postoperatively, the kyphosis angle was 75°. By 3 months after the operation, the patients breathing difficulty and back pain were completely relieved. The numbness in the lower extremities gradually diminished and was resolved by 6 months after the operation (ASIA E). The red arrow showed the orientation of the traction force

**Fig. 3:**
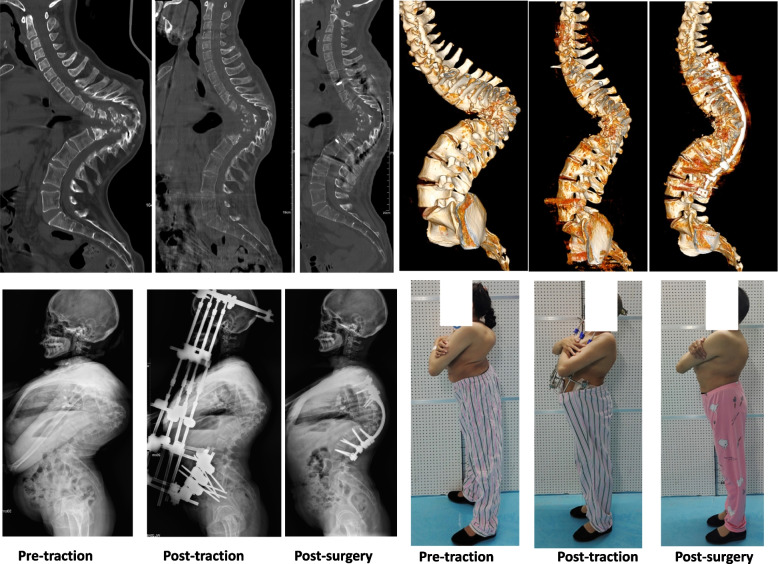
A 36-year-old female presented with numbness and weakness of both lower extremities for 2 months (ASIA C) and difficulty breathing after activity. Pulmonary function testing revealed a forced vital capacity of 0.96 L (37% of the expected value). CT revealed almost complete absence of the vertebral bodies from T5 to T12. HPT was applied for 12 weeks, and the weakness of both lower extremities was partially relieved (ASIA E). Pulmonary function testing revealed a forced vital capacity of 1.22 L (47% of the expected value). The kyphosis angle decreased from a pre-traction value of 148° to a post-traction value of 85°. Posterior fusion from T1 to L4 was performed, and the kyphosis angle was 73°

Yau et al. [26] reported a staged treatment of HPT combined with fusion and instrumentation for tuberculous kyphosis. However, the correction of kyphosis was only 28% with a 10% mortality rate. With the development of internal fixation techniques, the use of HPT gradually declined. More recently, some authors reported the use of traditional HPT to treat the tuberculous kyphosis and achieved effective correction [25, 27, 28]. Kim et al. [27] reported the case of a patient with tuberculous kyphosis and incomplete paraplegia. After 2 months traction, a posterior osteotomy and fusion was performed and the kyphotic deformity was corrected from 100° to 62°. Yu et al. [28] reported a case in which the kyphotic deformity was corrected from 180° to 30° after 10 months HPT and posterior fusion. Muheremu et al. [25] concluded that that HPT before osteotomy in patients with severe spinal kyphotic deformity due to spinal tuberculosis increases the efficacy and safety of surgical treatment. However, the aforementioned authors did not discuss the mechanism by which HPT improves surgical outcomes. In our study, we evaluated details of the spine and kyphosis by CT pre-traction and post-traction. Since spinal tuberculosis often involves and destroys multiple adjacent vertebral bodies, the angular kyphosis region is often completely fused and HPT has limited corrected capabilities in these fused areas. As such, it is important to know how HPT improves severe kyphosis prior to surgery. If the angular kyphosis region is completely fused it may appear as an abruption at the distal end of the kyphosis (Fig. 2) when HPT cannot reduce kyphotic deformity further. This can result in vascular and neurologic injury. However, in this study no patients had worsening of neurologic symptoms. This may be related to the stable, continuous traction provided by HPT. If the angular kyphosis is not completely fused, the kyphosis region can “unfold” due to the high corrective force of HPT (Fig. 3). In our prior study [15], the correction of kyphosis obtained by modified HPT was superior to the coronal correction (47.59% for kyphosis, 45.15% for scoliosis). This may be because the force of traction of the modified HPT device is primarily in front of the body, which makes it more effective for patients with severe kyphosis than scoliosis.

O’Brien et al. [14] described complications associated with HPT, including complications associated with the pins, injuries of the cervical spine, and nerve injuries and paraplegia. Pelvic pin loosening occurred in one patient in our study, but other serious complications were not observed. Moreover, different from the traditional HPT, the pelvic pins used in our HPT did not need to be drill through the iliac crest (Fig. 1), which could reduce the perforation of the intestine or injuries of other pelvic organs. O’Brien et al. reported that more than 50% of patients developed degeneration of the cervical spine when the traction was maintained for more than 3 months [14]. In our study, mean length of traction was 10.4 weeks, which was much shorter than 3 months and no degeneration of the cervical spine was observed. In our study, one patient (5.2%) developed neurologic symptoms during HPT, but they resolved after reducing the traction. Importantly, with our method HPT is lengthened while the patient is conscious, therefore any neurologic deficits can be observed immediately if they occur. There was only one major complication associated with the corrective surgery in this study. The pull-out of a proximal screw was noted at the 3-month follow-up in one patient. But no revision surgery was needed, and there was no progression of the screw pull-out during the following 2 years.

In the 1960s, O’Brien et al. [16] reported the use of HPT to correct various spinal deformities. Overall, they obtained an effective correction but the drawbacks of traditional HPT were obvious, such as a long period of hospitalization, a multi-stage process, and numerous complications. O’Brien et al. [14] reported that the average time a patient spends in HPT was 7.5 months, and that almost all patients developed some type of complication. Our modified HPT apparatus is different from the whole-ring pelvic frame of traditional HPT; the pelvic ring of our HPT device is a half-ring, and thus there are no pins around the posterior pelvis (Fig. 1). The modification helps reduce the discomfort and inconvenience of traditional HPT, and allows patients to sleep in a supine position, wear clothes, and move by themselves while still achieving continuous traction.

Although Kim et al. reported that HPT achieved effective correction when used to treat tuberculous kyphosis, the reports of Kim et al. and Yu et al. were case reports [27, 28]. Muheremu et al. [25] reported the results of 40 patients with tuberculous kyphosis treated with HPT; however, they only performed radiographic assessment. While our study showed good results with the use of our modified HPT device, there are limitations of our study. The standard protocol for treating severe scoliosis cases at our center is the procedure described in this report, this study was a retrospective review of our patients without comparison to a control group. Secondly, only 19 patients were included in the analysis. A such, in the future study of a larger number of patients is necessary to confirm the effectiveness and safety of our method.

## Conclusions

In conclusion, HPT is effective in the management of severe spinal kyphosis secondary to tuberculosis. Preoperative HPT can greatly reduce the kyphosis, and the need for corpectomy.

## Supplementary Information

**Additional file 1.**

**Additional file 2.** 

## Data Availability

The patients’ data were collected in the hospital. The datasets used and/or analyzed during the current study are available from the corresponding author on reasonable request.

## Abbreviations

HPT: Halo-pelvic traction
CT: Computed tomography
VAS: Visual analogue scale
SVA: Sagittal vertical axis
SRS-22: Scoliosis research society outcomes questionnaire
ODI: Oswestry Disability Index
PVCR: Posterior vertebral column resection
CWO: Closing opening wedge osteotomy
PSO: Pedicle subtraction osteotomy
