# Supplementary figures and images for: Two-stage treatment for severe spinal kyphotic deformity secondary to tuberculosis: halo-pelvic traction followed by a posterior-only approach correction
Source: BMC Musculoskelet Disord. 2022 Nov 18;23:991. doi: 10.1186/s12891-022-05974-7 (PMC9673369; doi:10.1186/s12891-022-05974-7)

## Slide 1
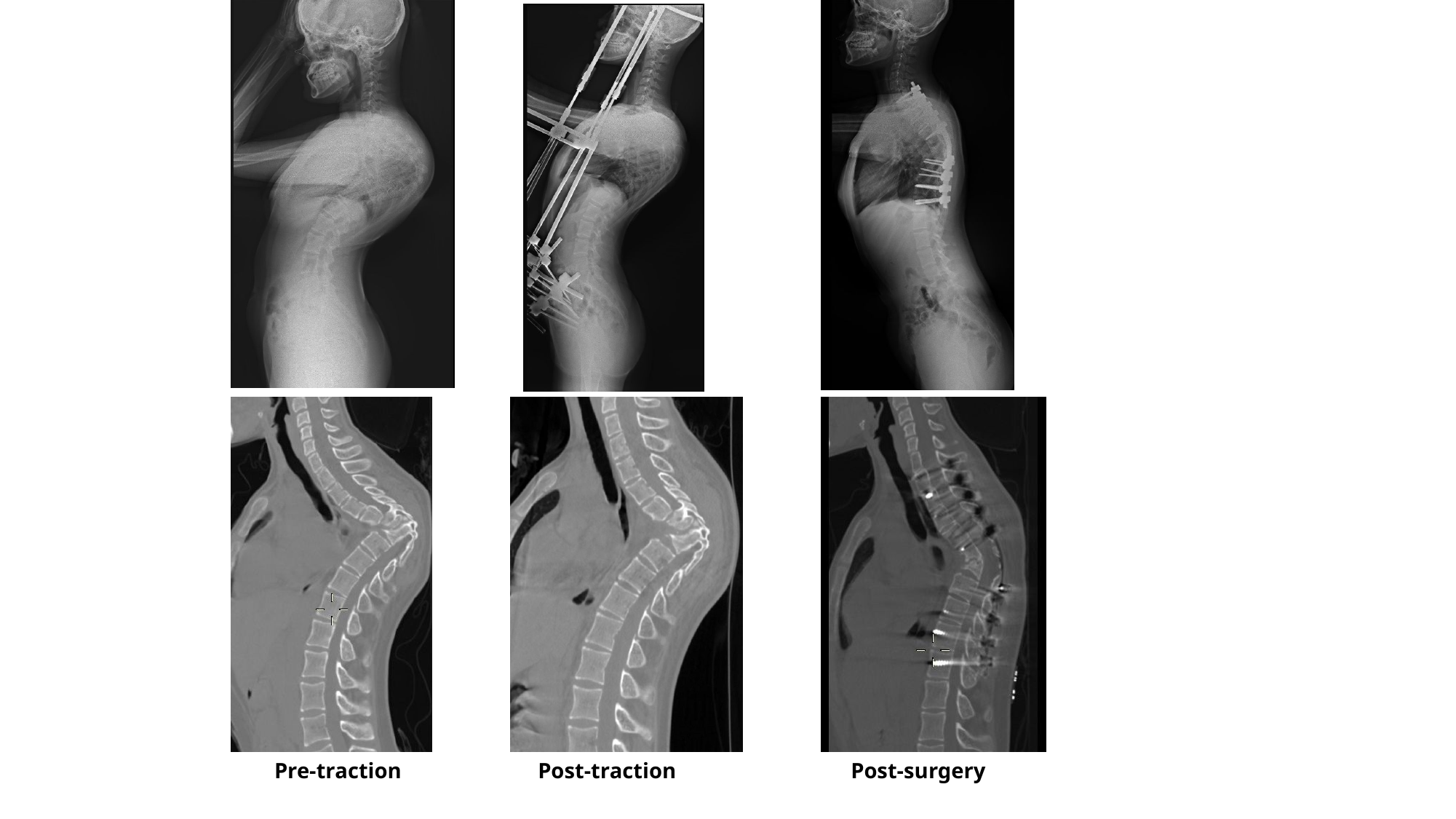

Pre-traction Post-traction Post-surgery

Supplement: Supplementary file 2 — Additional file 2. [file 12891_2022_5974_MOESM2_ESM.pptx]
